# Retraction Note: Assessing cardiorespiratory fitness of soccer players: is test specificity the issue?–a review

**DOI:** 10.1186/s40798-019-0217-9

**Published:** 2019-10-17

**Authors:** Monèm Jemni, Mohammad Shoaib Prince, Julien S. Baker

**Affiliations:** 1 Sport Science Program, College of Arts and Sciences, Qatar University, 1396 Al Tarfa, 2713, Doha, 2713 Qatar; 2 Institute of Clinical Exercise and Health Science, Applied Physiology Research Laboratory, School of Science and Sport, University of the West of Scotland, Hamilton, Lanarkshire ML3 OJB Scotland

**Correction to: Sports Med Open (2018) 4:28.**

**https://doi.org/10.1186/s40798-018-0134-3**

The editors have retracted this article [1] because it contains substantial text and figure overlap with the following articles [2–5]. The first author, Monèm Jemni, has not confirmed whether he agrees or disagrees with this retraction; the second and third authors, Mohammad Shoaib Prince and Julien S. Baker, agree with this retraction.
